# Patients’ transition experience and care from predialysis to dialysis: a theory-guided integrative review

**DOI:** 10.1186/s12882-025-04104-4

**Published:** 2025-04-08

**Authors:** Xuefei Wang, Ke Tian, Jin Hu, Shengqin Kang, Shunzhi Deng, Xueyan Gao, Yongzhen Mo

**Affiliations:** 1 Geriatric Hospital of Nanjing Medical University, Nanjing, Jiangsu China; 2 School of Nursing, Nanjing Medical University, Nanjing, Jiangsu China; 3 School of Nursing, Department of Medicine, Soochow University, Suzhou, Jiangsu China

**Keywords:** Advanced chronic kidney disease, Transition theory, Predialysis, Dialysis, Integrative review

## Abstract

**Background:**

A smooth transition to dialysis is essential for survival and quality of life in patients with advanced chronic kidney disease (CKD).

**Objective:**

To develop a transition framework for patients with advanced CKD based on the existing research and transition theory, which aims to illuminate patients’ transition experience and provide potential intervention strategies.

**Methods:**

An integrative review methodology was employed, with searches conducted in ten Chinese and English databases (PubMed, Web of Science, CINAHL, Embase, etc.). Articles were screened and selected based on predefined criteria independently by two authors, with reference lists of included studies reviewed for further studies. Data analysis followed the approach proposed by Whittemore and Knafl.

**Results:**

13 qualitative, 7 quantitative and 1 mixed methods articles were extracted and evaluated. This review develops the transition framework for patients with advanced CKD, including the concepts of transition nature, conditions, intervention strategies, and response patterns. It provides a comprehensive understanding of how personal, dialysis-related, interpersonal, community, and societal factors shape patients’ transition experiences and identifies actionable strategies to enhance transitional care.

**Conclusion:**

The transition of patients with advanced CKD from predialysis to dialysis is multiple and dynamic. Healthcare professionals should take into account diverse factors influencing this process and formulate tailored strategies to support patients in achieving a smooth and healthy transition.

**Clinical trial number:**

Not applicable.

**Supplementary Information:**

The online version contains supplementary material available at 10.1186/s12882-025-04104-4.

## Introduction

Chronic kidney disease (CKD) affects over 850 million people worldwide, with a global median prevalence of 9.5% [1, 2]. For patients with advanced CKD, defined by an estimated glomerular filtration rate (eGFR) below 25 ml/min/1.73 m², preparing for kidney replacement therapy (KRT), such as dialysis or transplantation, or conservative treatment, becomes essential [3, 4]. In particular, dialysis remains the most common form of KRT [5]. By 2021, approximately 3.8 million patients were receiving dialysis treatment [6]. The median annual cost was $38,339 per person for dialysis [2].

From predialysis to stable dialysis, patients go through a transition process from understanding and adapting to the disease and treatment mode, and most of them undergo several key stages, including the diagnosis, preparation (including dialysis mode selection and access establishment), and initiation of dialysis [4, 7]. This period is marked by challenges such as rapid health deterioration, changes in social roles, disruptions to daily life, and limited knowledge about disease management. These challenges may discourage timely dialysis, increasing the risk of unplanned emergency dialysis [8, 9]. Emergency dialysis, often initiated without mature dialysis access, significantly raises mortality rates and medical expenses [10].

In order to help patients facilitate a seamless and safe transition to the dialysis stage, many countries have published guidelines or consensus for managing predialysis kidney disease [11–16]. For instance, KDIGO 2024 clinical practice guideline for the evaluation and management of CKD, 2022 China Guidelines for peridialysis management of CKD provide structured recommendations for healthy transition to dialysis [11, 17]. Relevant research highlights that predialysis education and care can help prevent emergency dialysis, reduce complications, support shared decision-making, and alleviate negative emotions [18–20]. Although nephropathy management researchers have reached a consensus on the importance of patients’ healthy transition to dialysis, existing studies are still fragmented in exploring patients’ experience of dialysis transition and the anticipated benefits have not been fully realized [8, 21].

Meleis’ transition theory offers a valuable perspective for understanding and navigating individual healthy transition. This theory conceptualizes transitions as multifaceted processes influenced by four interrelated components: (1) the nature of transition (types, patterns, properties); (2) transition conditions (personal, community, and society); (3) intervention strategies; and (4) patterns of response (process and outcome indicators) [22–24]. Meleis’ transition theory has been widely used to explore transitions in contexts such as pregnancy, surgery, and cancer survivorship, and its systematicity provides an ideal framework for understanding the dialysis transition in advanced CKD patients [25–27].

To minimize the distress of patients face during their transition to dialysis, address their potential needs, help ensure a smooth progression to the dialysis stage, and reduce the risk of emergency dialysis while improving patient outcomes, this integrative review aims to address the current gap by achieving three main objectives: (1) synthesizing existing research on the transition from predialysis to dialysis; (2) developing a framework model on the basis of previous studies’ constituted concepts and modify it with classical transition theory into a holistic view for explaining patients’ transition experiences; and (3) proposing potential intervention strategies and policy recommendations to better support this population.

## Methods

This integrative review was guided by the five-stage framework by Whittemore and Knafl [28], including problem identification, literature search, data evaluation, data analysis, and presentations five stages. This review follows the Preferred Reporting Items for Systematic Reviews and Meta-Analyses (PRISMA) 2020 guidelines [29], and the review protocol was registered with PROSPERO [CRD 42024613874].

### Problem identification

The transition experience from predialysis to dialysis in patients with advanced CKD has not been systematically explained, and intervention strategies remain unclear.

### Literature search

#### Eligibility criteria

Articles were included if they focused on the experiences of patients with advanced CKD (eGFR < 25 ml/min/1.73m^2^) who transitioned to dialysis or explored measures facilitating a healthy transition. Exclusion criteria were: (1) patients on dialysis for more than 12 months; (2) conference abstracts, case reports or comments; (3) unavailable for full-text articles or inaccessible records; (4) publications not in Chinese or English.

#### Search strategy

Based on PICOS (Population, Intervention, Comparison, Outcome, and Study design) and PICoS (Population, Interest of Phenomena, Context, and Study design) framework, the search strategy used a combination of subject headings and keywords. The review conducted a comprehensive search across multiple databases, including PubMed, Web of Science, CINAHL, Embase, the Cochrane Library, the China National Knowledge Infrastructure (CNKI), Wanfang Data, VIP, Yiigle, China Biology Medicine disc. Records from each database’s inception until October 19, 2024, were included. Details of the search strategy are provided in Appendix 1.

### Data evaluation

The main information from each study was imported into Endnote. Titles and abstracts were initially screened, followed by a full-text review of studies meeting the eligibility criteria. Two researchers with expertise in kidney disease management and evidence-based medicine independently assessed study quality using the Mixed Methods Appraisal Tool (MMAT) [30]. The MMAT includes two initial screening questions and five methodological questions tailored to the study type. Each question was rated as “Yes” (green), “Unclear” (yellow), or “No” (red). Any discrepancies in evaluations were resolved through team discussion.

### Data analysis

Guided by Whittemore and Knafl’s integrative review framework [28], the analysis involved three iterative phases: (1) Data reduction: Findings from included studies were systematically categorized into Meleis’ transition theory components (nature, conditions, interventions, and response patterns). Key themes (e.g., “critical events” under transition nature) were inductively extracted and organized into thematic matrices to align with theoretical constructs. (2) Data comparison: Thematic clustering (e.g., consolidating “insurance barriers” into societal conditions) and frequency analysis were employed to identify patterns and contrasts across studies. Emergent themes (e.g., dialysis-related intrusiveness) were iteratively refined through researcher triangulation and team consensus to ensure conceptual coherence. (3) Conclusion verification: To validate theoretical propositions, qualitative themes (e.g., “unmet informational needs”) were mapped to quantitative outcomes (e.g., a 64% reduction in emergency dialysis rates associated with predialysis education, *P* < 0.001). Final themes underwent cross-validation against 30% of primary sources via member checking, minimizing interpretive bias and enhancing validity.

## Results

### Search results

**Fig. 1:**
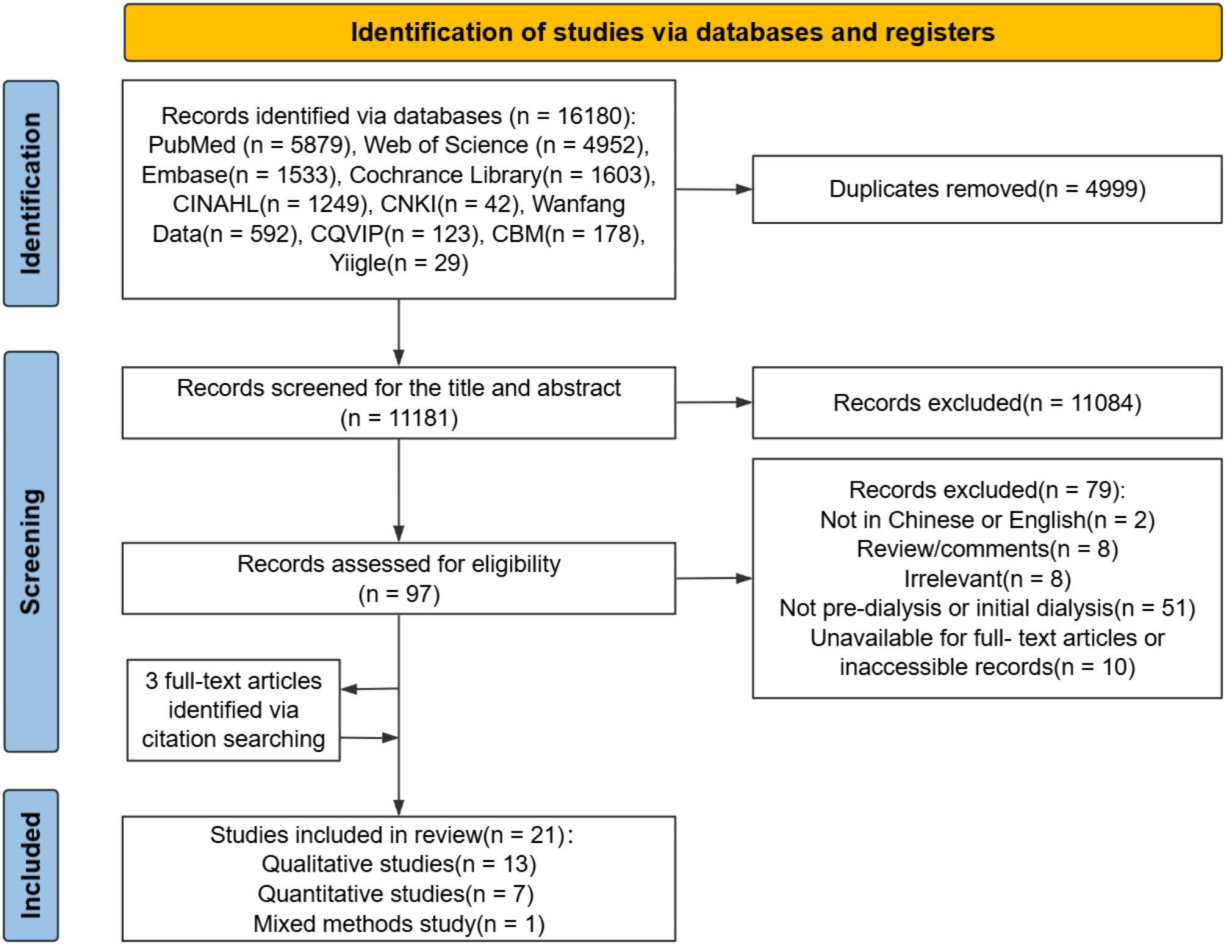
PRISMA flow diagram

The initial search identified 16,180 records. After removing duplicate records, 11,181 records were screened by title and abstract. Following this preliminary screening, 97 full-text articles were reviewed, of which 79 were excluded for not meeting the inclusion criteria. Three additional articles were identified through citation searching. A total of 21 studies were included. Figure 1 summarizes the process of the study identification, selection and screening of the articles.

### Evaluation of the literature

All 21 included studies were evaluated for methodological quality. Details regarding the quality assessment of each article can be found in Appendix 2. No studies were excluded solely on the basis of their methodological quality appraisal, ensuring a comprehensive understanding of the existing evidence base.

### Study characteristics

Of the 21 studies, 13 were qualitative [9, 31–42], 7 employed quantitative methods: 1 randomized controlled trial (RCT) [43], 1 quasi-experimental study [20], 4 retrospective cohort studies [18, 19, 44, 45], 1 prospective cohort study [46], and 1 mixed methods study [8]. The studies were conducted across 11 countries, including the United States, the United Kingdom, Australia, New Zealand, China, etc., and were published between 2005 and 2024. Among the included literatures, 3 Chinese-language publications and 18 English-language publications were analyzed. All non-English studies underwent translation and cross-verification by bilingual researchers to ensure accuracy in data extraction and interpretation. Participants primarily comprised patients with advanced CKD transitioning from predialysis to early dialysis (dialysis duration ≤ 12 months), with some studies incorporating family caregivers. Key characteristics of the included studies are summarized in Tables 1 and 2.

### Transition framework for patients with advanced CKD

**Fig. 2:**
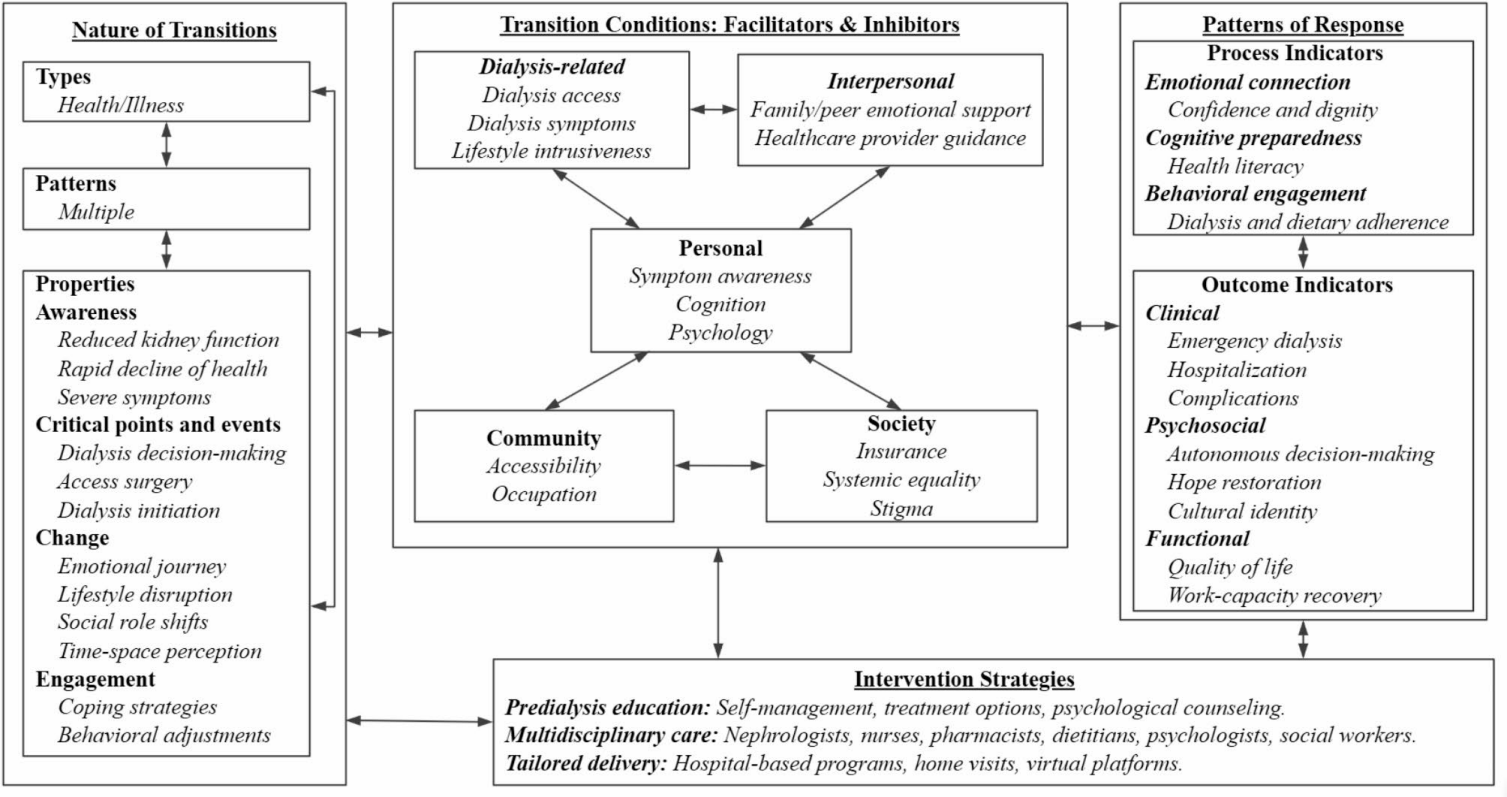
Transition framework for patients with advanced CKD from predialysis to dialysis

Using Meleis’ transition theory as a foundation [22], this review developed a comprehensive framework to understand the experiences of patients transitioning from predialysis to dialysis. The revised framework (Fig. 2) integrates key qualitative and quantitative findings, expanding the original theory to address dialysis-specific challenges and multidimensional influences. Organized into four interconnected domains—(1) transition nature, (2) facilitators and inhibitors, (3) intervention strategies, and (4) response patterns—the framework adapts Meleis’ foundational components (bolded) to advanced CKD. Novel themes (italicized), such as dialysis-related barriers (e.g., access surgery concerns) and interpersonal facilitators (e.g., family/peer support), emerged from synthesized evidence to reflect patients’ lived experiences, thereby bridging theoretical constructs with empirical realities in this critical health transition.

**Table 1:** Characteristics of included qualitative studies (*n* = 13)

Author (year)	Country	Study type	Participants	Key findings	Framework alignment	Extended themes
Iles-Smith (2005)	UK	Qualitative	10 predialysis patients	1. Perceptions of treatment: Lack of clear expectations, viewing dialysis as inconvenient with fatalism. 2. Information challenges: Incomplete information and comprehension barriers.	Transition conditions: Personal (negative psychology)	Transition conditions: Dialysis-related (intrusiveness)
Mitchell (2009)	UK	Qualitative	10 dialysis patients ≤ 6 months	1. Preparation: Importance of education and choice. 2. Cognitive style: Positive reappraisal and optimism. 3. Social support: Family, friends, and peer support.	- Transition nature: Critical events (dialysis decision-making) - Transition conditions: Personal (positive cognition)	Transition conditions: Interpersonal (support)
Lai (2012)	Singapore	Qualitative	13 HD patients ≤ 6 months	1. Emotional distress: Burden, loss of purpose, fear of death. 2. Treatment concerns: Symptoms, intrusiveness, access-site issues. 3. Social support: Informational and instrumental support.	- Transition nature: Change (negative emotions) - Transition conditions: Personal (negative psychology)	Transition conditions: Dialysis-related (symptoms, access concerns, intrusiveness); Interpersonal (support)
Yu (2013)	China	Qualitative	25 diabetic patients undergoing initial HD	1. Disease stages: Asymptomatic to burden/shock. 2. Coping strategies: Spiritual comfort and acceptance.	Transition nature: Awareness (symptom recognition); Change (emotional shifts); Engagement (coping)	—
Monaro (2014)	Australia	Qualitative	11 HD patients ≤ 3 months and 5 caregivers	1. Emotional impact: Shock, grief, loss of spontaneity. 2. Role changes: Family role reframing and social disconnection.	Transition nature: Change (emotional and social roles)	—
Cervantes (2017)	USA	Qualitative	20 undocumented advanced CKD patients	1. Distressing symptoms: Unpredictable emergent HD access. 2. Social consequences: Family and societal impacts. 3. Healthcare perceptions: Systemic inequities.	- Transition nature: Awareness (severe symptoms); Change (emotion) - Transition conditions: Society (systemic inequities)	Transition conditions: Interpersonal (family and healthcare support)
Gullick (2017)	Australia	Qualitative	11 dialysis patients ≤ 3 months and 5 caregivers	1. Compartmentalization: Dialysis vs. non-dialysis days. 2. Temporal experience: Adapting to rigid schedules.	Transition nature: Change (time-space constraints); Engagement (behavioral adjustments)	Transition conditions: Dialysis-related (intrusiveness)
Henry (2017)	USA	Qualitative	168 patients undergoing initial dialysis and 16 caregivers	1. RRT preparation: Fear and ambivalence. 2. Patient perspectives: Suggestions to improve RRT experience.	- Transition nature: Critical events (access surgery, initiation) - Transition conditions: Community (accessibility)	Transition conditions: Interpersonal (healthcare provider guidance)
Lovell (2017)	New Zealand	Qualitative	17 predialysis or dialysis ≤ 6 months patients	1. Decision-making: Delaying dialysis due to independence loss. 2. Dialysis imperative: Reluctant acceptance.	- Transition nature: Awareness (health decline); Critical events (dialysis decision-making) - Transition conditions: Personal (negative psychology)	Transition conditions: Dialysis-related (intrusiveness)
Walker (2017)	New Zealand	Qualitative	13 predialysis or dialysis ≤ 12 months patients	1. Stigma: Confronting kidney disease stigma. 2. Cultural identity: Maintaining identity during treatment. 3. Decision-making: Building supportive relationships.	- Transition nature: Change (negative emotions) - Transition conditions: Society (stigma) - Response patterns: Outcome (cultural identity)	Transition conditions: Interpersonal (family, peer, and healthcare support)
Nilsson (2019)	Sweden	Qualitative	5 unplanned HD patients	1. Health decline: Rapid deterioration leading to unplanned HD. 2. Acceptance: Difficult adjustment. 3. Social support: Key supporters.	Transition nature: Awareness (health/kidney function decline); Critical events (unplanned initiation); Change (emotion); Engagement (adjustment)	Transition conditions: Interpersonal (support)
Wang (2022)	China	Qualitative	15 peri-dialysis HD patients	1. Psychological experience: Negative emotions and coping shifts. 2. Disease cognition: Insufficient understanding.	- Transition nature: Change (negative emotions); Engagement (coping) - Transition conditions: Personal (insufficient understanding, negative psychology); Society (stigma)	—
Mehta (2024)	USA	Qualitative	20 dialysis patients ≤ 3 months	1. Emotional overwhelm: Shock and fear. 2. Coping strategies: Sense-making and moving forward.	Transition nature: Change (emotion); Engagement (coping)	Transition conditions: Dialysis-related (symptoms, intrusiveness); Interpersonal (healthcare guidance)

**Table 2:** Characteristics of included quantitative and mixed-methods studies (*n* = 8)

Author (year)	Country	Study design	Participants	Key findings	Framework alignment	Extended themes
Ma (2010)	China	Non-RCT	150 PD-eligible patients	Predialysis education improved adherence (82.1% vs. 41.7%, *P* < 0.001) and work capacity recovery (68.0% vs. 38.9%, *P* < 0.001).	- Response patterns: Process (adherence); Outcome (work capacity recovery) - Intervention strategies	—
Cho (2012)	South Korea	Retrospective cohort	1218 advanced CKD patients	Multidisciplinary predialysis education reduced unplanned dialysis (8.7% vs. 24.2%, *P* < 0.001), hospital days (2.16 vs. 5.05 days/year, *P* = 0.024), and cardiovascular events (2.7% vs. 9.4%, *HR* = 0.24, *P* = 0.017).	- Response patterns: Outcome (unplanned dialysis, hospitalization, complications) - Intervention strategies	—
Fishbane (2017)	USA	RCT	130 advanced CKD patients	Home visits reduced hospitalization rates (0.61 vs. 0.92, *RR* = 0.66, *P* = 0.04).	- Response patterns: Outcome (hospitalization) - Intervention strategies	—
Schanz (2017)	Germany	Retrospective cohort	336 new dialysis patients	In-hospital education increased free dialysis choice (28.5–62.7%, *P* < 0.0001) and PD utilization (16.6–27.6%, *P* = 0.02).	- Response patterns: Outcome (autonomous decision-making) - Intervention strategies	—
Shi (2019)	China	Retrospective cohort	233 advanced CKD patients	Hierarchical management reduced RRT use (39.6% vs. 53.5%, *P* = 0.037) and temporary catheterization (77.4% vs. 96.7%, *P* = 0.003).	- Response patterns: Outcome (unplanned dialysis) - Intervention strategies	—
Kaiser (2020)	Israel	Prospective cohort	98 advanced CKD patients	Virtual CKD care improved knowledge levels (52.0–94.0%, *P* < 0.001) and home dialysis interest (36.0–68.0%, *P* = 0.047).	- Response patterns: Process (health literacy) - Intervention strategies	—
Cervantes (2021)	USA	Mixed- methods	26 undocumented patients transitioning from emergency to scheduled HD	1. Anxiety and burden: Navigating care changes. 2. Outcomes: Restored hope and humanity. 3. Health gains: Quality-of-life improvements (*P* < 0.001) and symptom relief (*P* < 0.05).	- Transition nature: Change (negative emotions) - Transition conditions: Personal (negative psychology); Community (occupation); Society (stigma, insurance) - Response patterns: Process (rebuilding confidence, restoring dignity); Outcome (hope restoration, improvement in quality of life)	Transition conditions: Dialysis-related (symptoms)
Hundemer (2023)	Canada	Retrospective cohort	1070 advanced CKD patients	Social determinants (education: *OR* = 1.71, 95% *CI* = 1.09–2.69; employment: *OR* = 1.85, 95% *CI* = 1.18–2.92; marital status: *OR* = 1.44, 95% *CI* = 1.07–1.93) influenced transition to kidney failure.	Transition conditions: Community (occupation)	Transition conditions: Interpersonal (healthcare support)

#### Nature of the transition

The transition from predialysis to dialysis involves multiple patterns of health-to-illness experiences, shaped by several key properties. Awareness is the trigger for the beginning of the process, and patients often realize that the transition is about to begin when they notice a gradual decline in kidney function, accompanied by worsening health and the onset of related symptoms [9, 34, 36, 39]. Immediately following, patients tend to experience several critical points and events, such as dialysis decision-making, access surgery, and dialysis initiation [9, 38, 39]. Patients must adapt to changes in lifestyle, social roles, sense of time and space, progressing through phases of shock, fear, helplessness, acceptance, and reconciliation [8, 9, 33–35, 37, 38, 40–42]. Moreover, coping and adjustment strategies, such as medication adherence and dietary control, are integral to navigating this transition [9, 34, 41, 42].

#### Facilitators and inhibitors of transition

The transition in patients with advanced CKD involves multiple dimensions of facilitators and inhibitors, and is further divided into personal, dialysis-related, interpersonal, community, and social dimensions.

##### Personal

Symptom awareness accelerated transition, while poor cognition and negative psychological delayed a patient’s healthy transition [8, 31–33, 39, 41].

##### Dialysis-related

Dialysis related factors are unique to patients with advanced CKD, mainly reflected in the concern of dialysis access surgery and dialysis-related symptoms [8, 31, 38, 42]. In particular, the intrusion of dialysis on life will also affect patients’ engagement in treatment [33].

##### Interpersonal

Practical and emotional support from family, peer patients, and healthcare professionals gives patients the strength to cope with the challenges of the disease [9, 32, 33, 38–40, 42, 45]. Quantitative studies highlight that social determinants (e.g., marital status: *OR* = 1.44, 95% *CI* = 1.07–1.93) significantly influence transition outcome [45].

##### Community

Due to the long-term nature of dialysis and its relatively fixed location, patients have a demand for dialysis accessibility and job security [8, 38].

##### Society

Medical insurance coverage is a critical determinant of transitional care outcomes [8]. To illustrate, medicaid expansion policies at the state level significantly improved quality-of-life metrics (*P* < 0.001), demonstrating the profound societal impact of equitable healthcare access [8]. While limited in scope, systemic equality initiatives—such as standardized dialysis eligibility criteria—have been empirically linked to timely treatment initiation in emerging studies [36]. Moreover, cultural stigma is perceived as a social problem that can delay treatment [8, 40, 41].

#### Intervention strategies

Effective interventions to facilitate the smooth transition emphasize multidisciplinary collaboration care. These include predialysis education on self-management, treatment options and psychological counseling [18, 20, 43, 44, 46]. Interventions are delivered through hospital, home-based, and virtual platforms, ensuring comprehensive patient support throughout the transition process [19, 43, 46].

#### Patterns of response

Health transition indicators require continuous assessment throughout the transitional journey, rather than solely at its endpoint [22]. To operationalize this principle, our framework adopts a dual measurement approach: process evaluation metrics (tracking transitional dynamics) and outcome evaluation parameters (measuring endpoint achievements). Both dimensions were systematically derived through thematic synthesis of evidence from included studies, ensuring alignment with transitional theory while capturing empirical realities of dialysis initiation.

##### Process indicators

Process indicators capture dynamic adaptations during the transitional period across three interconnected domains: emotional connection, cognitive preparedness, and behavioral engagement. Qualitative studies emphasized the restorative role of emotional connections in rebuilding patients’ confidence and dignity [8]. In the cognitive domain, structured predialysis education programs demonstrated a 42% improvement in health literacy (*P* < 0.01), equipping patients with critical knowledge for informed decision-making [46]. Behavioral engagement, such as compliance with dialysis schedules, significantly reduced unplanned hospitalizations (*RR* = 0.66, *P* = 0.04), underscoring the clinical impact of routine stabilization [43].

##### Outcome indicators

Outcome indicators validate the effectiveness of transitional care across clinical, psychosocial, and functional dimensions. Clinically, multidisciplinary interventions reduced emergency dialysis rates (8.7% vs. 24.2%, *P* < 0.001) [18]. Psychosocially, qualitative studies identified cultural identity and restored hope as novel markers of successful transitions [8, 40]. Quantitative analyses concurrently demonstrated a 34.2% enhancement in autonomous decision-making rates (*P* < 0.0001) through hospital-based educational interventions [19]. Functionally, improvements in quality-of-life metrics and work capacity recovery indicated successful patient reintegration into societal roles [8, 20].

## Discussion

To our knowledge, this is the first review to apply transition theory to systematically examine the experiences of patients with advanced CKD transitioning from predialysis to dialysis, and to propose evidence-based intervention strategies. It provides an evidence-based framework comprising interrelated structures and propositions to better understand the transition process and inform intervention strategies. Three main points are discussed, namely (1) the nature and properties of transition, (2) personal and environmental conditions that facilitate or hinder progress toward achieving a healthy transition, and (3) effective intervention strategies to support patients during this critical period.

The transition from predialysis to stable dialysis is a multiple and dynamic process. A decline in renal function and the onset of symptoms often serve as triggers for initiating this transition. Critical events, such as dialysis decision-making, access surgery, and dialysis initiation, are pivotal for a successful transition [9, 38, 39]. Unlike the gradual progression of earlier stages of CKD, the advanced stages involve irreversible disease progression and continuous treatment dependence, the emotional journey of patients during this phase is variable. Fear is a high-frequency word mentioned by patients [8, 34, 38, 40]. This is consistent with findings in cancer patients [47]. The sources of fear are often multiple, such as illness, treatment, life, role, etc., which boils down to the fear of the unknown and uncertainty [38].

Dialysis is the most common treatment for patients with advanced CKD. Unlike treatments for other chronic diseases, advanced CKD requires a rigid dialysis schedule—typically 2–3 sessions per week—which significantly disrupts patients’ daily lives. Beyond the behavioral adaptations required to manage the disease, patients must navigate substantial changes in lifestyle, social roles and spatiotemporal perception. The invasive nature of dialysis often contributes to delays in treatment initiation [33, 37]. Furthermore, it was found that the factors influencing the healthy transition of patients with advanced CKD are multi-dimensional. Symptom awareness is an important signal that affects whether a patient transitions or not, which has been explored mainly in cancer population [48]. Previous studies have revealed that lower overall symptom awareness is correlated with poorer cancer survival, though more research is needed to examine the mechanisms through which awareness has its effects [49, 50]. As one advanced CKD patient stated: “it was only when the symptoms appeared that the reality about the need for dialysis materialised”. Unfortunately, limited symptom awareness often leaves patients unprepared in physical and mental, leading to emergency dialysis and worse prognoses.

The transition from predialysis to formal dialysis is often a ternary process involving the patient, family, and healthcare provider, with the common goal of participating stakeholders that patients can make a healthy transition after discharge in line with established goals or receive support in a problematic transition [51, 52]. Practical and emotional support from families plays a crucial role in ensuring a smooth transition, as patients lacking such support are more likely to refuse dialysis [39]. Informational support from healthcare providers is equally essential, enabling shared decision-making, which serves as the cornerstone for initiating dialysis and facilitating a healthy transition [53, 54]. However, it is important to note that decisions are multifaceted, influenced by symptoms, laboratory trajectories, patient preferences, and the cost and availability of treatment [55].

Healthcare policies critically shape dialysis access for undocumented immigrants. Cervantes et al. [36]revealed that in most U.S. states, undocumented patients with kidney failure are restricted to emergency dialysis only during life-threatening crises (e.g., hyperkalemia), a policy that compels some individuals to intentionally consume high-potassium foods to meet eligibility criteria—thereby exacerbating emergency dialysis rates. In contrast, states such as California, New York, and Colorado have expanded medicaid coverage to include scheduled dialysis, significantly reducing emergency hospitalizations and associated costs. For example, Colorado’s 2019 policy reform not only saved $19 million in its inaugural year but also improved patients’ quality of life by enabling consistent care [8]. Despite these advancements, persistent systemic barriers—including transportation inequities and fragmented care coordination—underscore the urgent need for federal policies to standardize dialysis access nationwide. Future reforms must prioritize patient-centered solutions, such as structural support and culturally sensitive care models, to address sociocultural determinants of health equity.

Education is a primary strategy for preparing patients for dialysis [56]. Quantitative studies demonstrate that structured predialysis education reduces emergency dialysis starts and hospitalizations, which corroborates qualitative findings highlighting patients’ unmet informational needs [18, 31, 40, 43]. This convergence underscores that educational interventions addressing psychological uncertainty simultaneously improve clinical outcomes and empower patients to navigate decision-making with greater confidence. For instance, process indicators such as behavioral engagement and emotional connection were enhanced through educational interventions, while outcome indicators—including reduced emergency dialysis rates and improved quality of life—demonstrated their long-term efficacy [8, 18].

This review highlights several implications for clinical practice and policy. First, healthcare providers must identify the critical events in the transition process and assess patients’ readiness for dialysis, considering physical, psychological, informational, familial, and practical dimensions. Second, due to the possible adverse health outcomes caused by the uncertainty of symptom awareness, future efforts should prioritize the development of standardized symptom monitoring tools and data-driven follow-up systems. Integrating these tools into health management platforms could facilitate early intervention and improve patient outcomes. Finally, healthcare systems should strive to enhance accessibility to dialysis facilities while aligning with local environmental or policy constraints. Such efforts could help normalize patients’ daily routines and support their reintegration into social roles, thereby mitigating the disruptive impact of dialysis.

Meleis’ transition theory provided a comprehensive lens to systematically analyze the multifaceted journey of patients transitioning from predialysis to dialysis. The framework’s emphasis on dynamic processes and multilevel influences aligned closely with the lived experiences of advanced CKD patients, who navigate physiological decline, psychosocial upheaval, and healthcare system complexities. Two pivotal advancements are underscored: (1) Integration of dialysis-specific factors: The inclusion of dialysis-related barriers (e.g., access surgery concerns, treatment intrusiveness) addresses gaps in Meleis’ original theory, which lacked disease-specific contextualization; (2) Balanced emphasis on qualitative and quantitative insights: While qualitative studies richly describe emotional and social dynamics, quantitative findings provide empirical validation.

While this review advances a theoretically informed framework for understanding transitions in advanced CKD, several limitations warrant consideration. First, the majority of included studies—both qualitative and quantitative—lacked explicit grounding in established theoretical frameworks, potentially limiting their conceptual rigor and generalizability. This gap underscores the need for future research to anchor study designs in mature theories, such as transition theory, to enhance methodological coherence. Second, while qualitative studies provided rich insights into patient experiences, the scarcity of RCTs restricts causal inferences about intervention efficacy. Nevertheless, the triangulation of quantitative findings with qualitative themes strengthened validity through methodological convergence. Third, few studies holistically examined patient outcomes across the transition continuum, resulting in fragmented evidence on process indicators and outcome metrics. Additionally, most of the included studies were conducted in high-income countries, and differences in geographic and cultural diversity may limit the applicability of the framework to underserved populations.

## Conclusions

This review presents a theoretical model to explain the transition experiences of patients with advanced CKD as they move from predialysis to dialysis. The model identifies factors that facilitate or hinder the transition across individual, dialysis-related, interpersonal, community, and societal levels. By offering a structured framework, the model provides a foundation for developing targeted interventions aimed at supporting a smoother and healthier transition. Future studies should build upon this framework to further investigate the multifaceted nature of the transition process and evaluate the effectiveness of intervention strategies.

## Electronic supplementary material

Below is the link to the electronic supplementary material.

Supplementary Material 1

Supplementary Material 2

## Data Availability

No datasets were generated or analysed during the current study.
