# Supplementary material for: Patients’ transition experience and care from predialysis to dialysis: a theory-guided integrative review
Source: BMC Nephrol. 2025 Apr 8;26:182. doi: 10.1186/s12882-025-04104-4 (PMC11980101; doi:10.1186/s12882-025-04104-4)
Supplement: Supplementary file 1 — Supplementary Material 1 [file 12882_2025_4104_MOESM1_ESM.docx]

**Appendix 1 Search Strategy**

**PICOS and PICoS framework**

| **PICOS** | | **PICoS** | |
| --- | --- | --- | --- |
| **P (population)** | advanced CKD patients on pre-dialysis and initial dialysis | **P (population)** | advanced CKD patients on pre-dialysis or initial dialysis |
| **I (intervention)** | interventions aimed at facilitating a smooth transition to dialysis in patients with advanced CKD | **I (interest of phenomena)** | patients' experiences |
| **C (comparison)** | usual care | **Co(context)** | transition from predialysis to dialysis |
| **O (outcomes)** | smooth transition |  |  |
| **S (study design)** | quantitative studies (randomized controlled trials, non-randomized controlled trials, quasi-experimental studies, cohort studies, and cross-sectional studies) | **S (study design)** | qualitative studies and mixed methods studies |

**Search Strategy for Each Database**

| **PubMed** | | |
| --- | --- | --- |
| #1 | ("Patients"[Mesh]) OR ("Inpatients"[Mesh]) OR ("Outpatients"[Mesh]) OR ("Adult"[Mesh]) OR (Patient*[TIAB]) OR (Inpatient*[TIAB]) OR (Outpatient*[TIAB]) OR (Adult*[TIAB]) OR (Client*[TIAB]) | 13,429,220 |
| #2 | ("Kidney Failure, Chronic"[Mesh]) OR ("Uremia"[Mesh]) OR (Chronic Renal Failure[TIAB]) OR (End Stage Kidney Disease*[TIAB]) OR (ESKD[TIAB]) OR (ESRD[TIAB]) OR (End Stage Renal Disease*[TIAB]) OR (End-Stage Renal Failure[TIAB]) OR (Chronic Kidney Failure[TIAB]) OR (Advanced Chronic Renal Insufficien*[TIAB]) OR (Advanced Chronic Kidney Insufficien*[TIAB]) OR (Advanced Chronic Kidney Disease*[TIAB]) OR (Advanced Chronic Renal Disease*[TIAB]) OR (Uremia*[TIAB]) | 166,201 |
| #3 | ("Renal dialysis"[Mesh]) OR ("dialysis"[Mesh]) OR ("Peritoneal dialysis"[Mesh]) OR ("Renal Replacement Therapy"[Mesh]) OR (Renal dialysis[TIAB]) OR (dialysis, Renal[TIAB]) OR (Hemodialysis[TIAB]) OR (Peritoneal dialysis[TIAB]) OR (dialysis, Extracorporeal[TIAB]) OR (Extracorporeal dialysis[TIAB]) OR (Renal replacement therap*[TIAB]) OR (predialysis[TIAB]) OR (peridialysis[TIAB]) | 297,310 |
| #4 | (Transfer*[TIAB]) OR(transit*[TIAB]) OR (Start*[TIAB]) OR (Begin*[TIAB]) OR (Initia*[TIAB]) OR (Go through[TIAB]) | 3,980,869 |
| #5 | (Experience*[TIAB]) OR (Life experience*[TIAB]) OR (Life change event*[TIAB]) OR (Life course*[TIAB]) OR (program[TIAB]) OR (manage*[TIAB]) OR (Intervention*[TIAB]) OR (care[TIAB]) | 5,760,646 |
| #6 | (#1 and #2) and (#3 and #4) and #5 | 5,879 |
| **Web of Science** | | |
| #1 | TS=(Patient* OR Inpatient* OR Outpatient* OR Adult* OR Client*) | 7,793,341 |
| #2 | TS=(Chronic Renal Failure OR End Stage Kidney Disease* OR ESKD OR ESRD OR End Stage Renal Disease* OR End-Stage Renal Failure OR Chronic Kidney Failure OR Advanced Chronic Renal Insufficien* OR Advanced Chronic Kidney Insufficien* OR Advanced Chronic Kidney Disease* OR Advanced Chronic Renal Disease* OR Uremia*) | 107,887 |
| #3 | TS=(Renal dialysis OR dialysis, Renal OR Hemodialysis OR Peritoneal dialysis OR dialysis, Extracorporeal OR Extracorporeal dialysis OR Renal replacement therap* OR predialysis OR peridialysis) | 130,042 |
| #4 | TS=(Transfer* OR transit* OR Start* OR Begin* OR Initia* OR Go through) | 6,218,629 |
| #5 | TS=(Experience* OR Life experience* OR Life change event* OR Life course* OR program OR manage* OR Intervention* OR care) | 6,365,264 |
| #6 | (#1 and #2) and (#3 and #4) and #5 | 4,952 |
| **Embase** | | |
| #1 | patient'/exp OR 'patient' OR 'adult'/exp OR 'adult' OR 'patient*':ti,ab OR 'inpatient*':ti,ab OR 'outpatient*':ti,ab OR 'adult*':ti,ab OR 'client*':ti,ab | 19,221,520 |
| #2 | end stage renal disease'/exp OR 'uremia'/exp OR 'End Stage Kidney Disease*':ti,ab OR 'ESKD':ti,ab OR 'ESRD':ti,ab OR 'End Stage Renal Disease*':ti,ab OR 'End-Stage Renal Failure':ti,ab OR 'Chronic Kidney Failure':ti,ab OR 'Chronic Renal Failure':ti,ab OR 'Advanced Chronic Renal Insufficien*':ti,ab OR 'Advanced Chronic Kidney Insufficien*':ti,ab OR 'Advanced Chronic Kidney Disease*':ti,ab OR 'Advanced Chronic Renal Disease*':ti,ab OR 'Uremia*':ti,ab | 176,604 |
| #3 | dialysis'/exp OR 'Renal dialysis':ti,ab OR 'dialysis, Renal':ti,ab OR 'Hemodialysis':ti,ab OR 'Peritoneal dialysis':ti,ab OR 'dialysis, Extracorporeal':ti,ab OR 'Extracorporeal dialysis':ti,ab OR 'Renal replacement therap*':ti,ab OR 'predialysis':ti,ab OR 'peridialysis':ti,ab | 306,944 |
| #4 | Transfer':ti,ab OR 'transit':ti,ab OR 'Start':ti,ab OR 'Begin':ti,ab OR 'Initia':ti,ab OR 'Go through':ti,ab | 1,040,534 |
| #5 | Experience*':ti,ab OR 'Life experience*':ti,ab OR 'Life change event*':ti,ab OR 'Life course*':ti,ab OR 'program':ti,ab OR 'manage*':ti,ab OR 'Intervention*':ti,ab OR 'care':ti,ab | 7,802,672 |
| #6 | (#1 and #2) and (#3 and #4) and #5 | 1,533 |
| **Cochrance Library** | | |
| #1 | MeSH descriptor: [Patients] explode all trees | 4,561 |
| #2 | MeSH descriptor: [Adult] explode all trees | 624,990 |
| #3 | MeSH descriptor: [Inpatients] explode all trees | 1,681 |
| #4 | (Patient* OR Inpatient* OR Outpatient* OR Adult* OR Client*):ti,ab,kw | 1,602,827 |
| #5 | MeSH descriptor: [Kidney Failure, Chronic] explode all trees | 6,113 |
| #6 | MeSH descriptor: [Uremia] explode all trees | 575 |
| #7 | (Chronic Renal Failure OR End Stage Kidney Disease* OR ESKD OR ESRD OR End Stage Renal Disease* OR End-Stage Renal Failure OR Chronic Kidney Failure OR Advanced Chronic Renal Insufficien* OR Advanced Chronic Kidney Insufficien* OR Advanced Chronic Kidney Disease* OR Advanced Chronic Renal Disease* OR Uremia*):ti,ab,kw | 22,561 |
| #8 | MeSH descriptor: [dialysis] explode all trees | 304 |
| #9 | MeSH descriptor: [Renal dialysis] explode all trees | 7,450 |
| #10 | MeSH descriptor: [Renal Replacement Therapy] explode all trees | 12,755 |
| #11 | (Renal dialysis OR dialysis, Renal OR Hemodialysis OR Peritoneal dialysis OR dialysis, Extracorporeal OR Extracorporeal dialysis OR Renal replacement therap* OR predialysis OR peridialysis):ti,ab,kw | 24,398 |
| #12 | (Transfer* OR transit* OR Start* OR Begin* OR Initia* OR Go through):ti,ab,kw | 336,447 |
| #13 | (Experience* OR Life experience* OR Life change event* OR Life course* OR program OR manage* OR Intervention* OR care):ti,ab,kw | 992,452 |
| #14 | ((#1 OR #2 OR #3 OR #4) AND (#5 OR #6 OR #7)) AND ((#8 OR #9 OR #10 OR #11) AND #12) AND #13 | 1,603 |
| **CINAHL Complete** | | |
| #1 | (MH "Patients+") OR (MH "Inpatients") OR (MH "Outpatients") OR (MH "Adult+") OR TI (Patient* OR Inpatient* OR Outpatient* OR Adult* OR Client*) OR AB (Patient* OR Inpatient* OR Outpatient* OR Adult* OR Client*) | 3,636,118 |
| #2 | (MH "Kidney Failure, Chronic+") OR (MH "Uremia+") OR TI (Chronic Renal Failure OR End Stage Kidney Disease* OR ESKD OR ESRD OR End Stage Renal Disease* OR End-Stage Renal Failure OR Chronic Kidney Failure OR Advanced Chronic Renal Insufficien* OR Advanced Chronic Kidney Insufficien* OR Advanced Chronic Kidney Disease* OR Advanced Chronic Renal Disease* OR Uremia*) OR AB (Chronic Renal Failure OR End Stage Kidney Disease* OR ESKD OR ESRD OR End Stage Renal Disease* OR End-Stage Renal Failure OR Chronic Kidney Failure OR Advanced Chronic Renal Insufficien* OR Advanced Chronic Kidney Insufficien* OR Advanced Chronic Kidney Disease* OR Advanced Chronic Renal Disease* OR Uremia*) | 34,467 |
| #3 | (MH "dialysis+") OR (MH "Peritoneal dialysis+") OR (MH "Renal Replacement Therapy+") OR TI (Renal dialysis OR dialysis, Renal OR Hemodialysis OR Peritoneal dialysis OR dialysis, Extracorporeal OR Extracorporeal dialysis OR Renal replacement therap* OR predialysis OR peridialysis) OR AB (Renal dialysis OR dialysis, Renal OR Hemodialysis OR Peritoneal dialysis OR dialysis, Extracorporeal OR Extracorporeal dialysis OR Renal replacement therap* OR predialysis OR peridialysis) | 51,808 |
| #4 | TI (Transfer* OR transit* OR Start* OR Begin* OR Initia* OR Go through) OR AB (Transfer* OR transit* OR Start* OR Begin* OR Initia* OR Go through) | 627,616 |
| #5 | TI (Experience* OR Life experience* OR Life change event* OR Life course* OR program OR manage* OR Intervention* OR care) OR AB (Experience* OR Life experience* OR Life change event* OR Life course* OR program OR manage* OR Intervention* OR care) | 2,288,442 |
| #6 | (S1 AND S2) AND (S3 AND S4) AND S5 | 1,249 |
| **CNKI** | | |
|  | SU%=((患者 + 病人) * (终末期肾病 + 终末期肾脏病 + 终末期肾脏疾病 + ESRD + ESKD + 晚期慢性肾脏病 + 晚期CKD + 慢性肾衰竭CKD5期 + 慢性肾脏病CKD5期 + 慢性肾脏病5期 + 肾衰竭 + 尿毒症)) AND SU%=((肾脏替代治疗 + 透析 + 透析前后 + 血液透析 + 腹膜透析 + HD + PD + 围透析期 + 围透析期护理 + 围透析期CKD + 透析前期 + 初始透析 + 透析前 + 透析前准备) * (过渡 + 开始 + 启动 + 首次 + 初次)) AND SU%=(经历 + 体验 + 感受 + 生活变化 + 管理 + 干预 + 支持) | 42 |
| **Wanfang Data** | | |
|  | 主题:(((患者 OR 病人) AND (终末期肾病 OR 终末期肾脏病 OR 终末期肾脏疾病 OR ESRD OR ESKD OR 晚期慢性肾脏病 OR 晚期CKD OR 慢性肾衰竭CKD5期 OR 慢性肾脏病CKD5期 OR 慢性肾脏病5期 OR 肾衰竭 OR 尿毒症)) AND ((肾脏替代治疗 OR 透析 OR 透析前后 OR 血液透析 OR 腹膜透析 OR HD OR PD OR 围透析期 OR 围透析期护理 OR 围透析期CKD OR 透析前期 OR 初始透析 OR 透析前 OR 透析前准备) AND (过渡 OR 开始 OR 启动 OR 首次 OR 初次)) AND (经历 OR 体验 OR 感受 OR 生活变化 OR 管理 OR 干预 OR 支持)) | 592 |
| **VIP** | | |
|  | T=(((患者 OR 病人) AND (终末期肾病 OR 终末期肾脏病 OR 终末期肾脏疾病 OR ESRD OR ESKD OR 晚期慢性肾脏病 OR 晚期CKD OR 慢性肾衰竭CKD5期 OR 慢性肾脏病CKD5期 OR 慢性肾脏病5期 OR 肾衰竭 OR 尿毒症)) AND ((肾脏替代治疗 OR 透析 OR 透析前后 OR 血液透析 OR 腹膜透析 OR HD OR PD OR 围透析期 OR 围透析期护理 OR 围透析期CKD OR 透析前期 OR 初始透析 OR 透析前 OR 透析前准备) AND (过渡 OR 开始 OR 启动 OR 首次 OR 初次)) AND (经历 OR 体验 OR 感受 OR 生活变化 OR 管理 OR 干预 OR 支持)) OR R=(((患者 OR 病人) AND (终末期肾病 OR 终末期肾脏病 OR 终末期肾脏疾病 OR ESRD OR ESKD OR 晚期慢性肾脏病 OR 晚期CKD OR 慢性肾衰竭CKD5期 OR 慢性肾脏病CKD5期 OR 慢性肾脏病5期 OR 肾衰竭 OR 尿毒症)) AND ((肾脏替代治疗 OR 透析 OR 透析前后 OR 血液透析 OR 腹膜透析 OR HD OR PD OR 围透析期 OR 围透析期护理 OR 围透析期CKD OR 透析前期 OR 初始透析 OR 透析前 OR 透析前准备) AND (过渡 OR 开始 OR 启动 OR 首次 OR 初次)) AND (经历 OR 体验 OR 感受 OR 生活变化 OR 管理 OR 干预 OR 支持)) | 123 |
| **China Biology Medicine disc(SinoMed)** | | |
|  | ((("肾功能衰竭, 慢性"[不加权:扩展]) OR "尿毒症"[不加权:扩展] OR "终末期肾病"[标题] OR "终末期肾脏病"[标题] OR "终末期肾脏疾病"[标题] OR "ESRD"[标题] OR "ESKD"[标题] OR "晚期慢性肾脏病"[标题] OR "晚期CKD"[标题] OR "慢性肾衰竭CKD5期"[标题] OR "慢性肾脏病CKD5期"[标题] OR "慢性肾脏病5期"[标题] OR "肾衰竭"[标题] OR "尿毒症"[标题] OR "终末期肾病"[摘要] OR "终末期肾脏病"[摘要] OR "终末期肾脏疾病"[摘要] OR "ESRD"[摘要] OR "ESKD"[摘要] OR "晚期慢性肾脏病"[摘要] OR "晚期CKD"[摘要] OR "慢性肾衰竭CKD5期"[摘要] OR "慢性肾脏病CKD5期"[摘要] OR "慢性肾脏病5期"[摘要] OR "肾衰竭"[摘要] OR "尿毒症"[摘要]) AND ("患者"[标题] OR "病人"[标题] OR "患者"[摘要] OR "病人"[摘要])) AND (("过渡"[标题] OR "开始"[标题] OR "启动"[标题] OR "首次"[标题] OR "初次"[标题] OR "过渡"[摘要] OR "开始"[摘要] OR "启动"[摘要] OR "首次"[摘要] OR "初次"[摘要]) AND (("肾透析"[不加权:扩展]) OR "肾脏替代治疗"[标题] OR "透析"[标题] OR "透析前后"[标题] OR "血液透析"[标题] OR "腹膜透析"[标题] OR "HD"[标题] OR "PD"[标题] OR "围透析期"[标题] OR "围透析期护理"[标题] OR "围透析期CKD"[标题] OR "透析前期"[标题] OR "初始透析"[标题] OR "透析前"[标题] OR "透析前准备"[标题] OR"肾脏替代治疗"[摘要] OR "透析"[摘要] OR "透析前后"[摘要] OR "血液透析"[摘要] OR "腹膜透析"[摘要] OR "HD"[摘要] OR "PD"[摘要] OR "围透析期"[摘要] OR "围透析期护理"[摘要] OR "围透析期CKD"[摘要] OR "透析前期"[摘要] OR "初始透析"[摘要] OR "透析前"[摘要] OR "透析前准备"[摘要])) AND (("经历"[摘要] OR "体验"[摘要] OR "感受"[摘要] OR "生活变化"[摘要] OR "管理"[摘要] OR "干预"[摘要] OR "支持"[摘要]) OR ("经历"[标题] OR "体验"[标题] OR "感受"[标题] OR "生活变化"[标题] OR "管理"[标题] OR "干预"[标题] OR "支持"[标题])) | 178 |
| **Yiigle** | | |
|  | (((标题=(患者 OR 病人)) OR (摘要=(患者 OR 病人))) AND ((标题=(终末期肾病 OR 终末期肾脏病 OR 终末期肾脏疾病 OR ESRD OR ESKD OR 晚期慢性肾脏病 OR 晚期CKD OR 慢性肾衰竭CKD5期 OR 慢性肾脏病CKD5期 OR 慢性肾脏病5期 OR 肾衰竭 OR 尿毒症)) OR (摘要=(终末期肾病 OR 终末期肾脏病 OR 终末期肾脏疾病 OR ESRD OR ESKD OR 晚期慢性肾脏病 OR 晚期CKD OR 慢性肾衰竭CKD5期 OR 慢性肾脏病CKD5期 OR 慢性肾脏病5期 OR 肾衰竭 OR 尿毒症)))) AND (((标题=(肾脏替代治疗 OR 透析 OR 透析前后 OR 血液透析 OR 腹膜透析 OR HD OR PD OR 围透析期 OR 围透析期护理 OR 围透析期CKD OR 透析前期 OR 初始透析 OR 透析前 OR 透析前准备)) OR (摘要=(肾脏替代治疗 OR 透析 OR 透析前后 OR 血液透析 OR 腹膜透析 OR HD OR PD OR 围透析期 OR 围透析期护理 OR 围透析期CKD OR 透析前期 OR 初始透析 OR 透析前 OR 透析前准备))) AND ((标题=(过渡 OR 开始 OR 启动 OR 首次 OR 初次)) OR (摘要=(过渡 OR 开始 OR 启动 OR 首次 OR 初次)))) AND ((标题=(经历 OR 体验 OR 感受 OR 生活变化 OR 管理 OR 干预 OR 支持)) OR (摘要=(经历 OR 体验 OR 感受 OR 生活变化 OR 管理 OR 干预 OR 支持))) | 29 |
