# Supplementary material for: Patients’ transition experience and care from predialysis to dialysis: a theory-guided integrative review
Source: BMC Nephrol. 2025 Apr 8;26:182. doi: 10.1186/s12882-025-04104-4 (PMC11980101; doi:10.1186/s12882-025-04104-4)
Supplement: Supplementary file 2 — Supplementary Material 2 [file 12882_2025_4104_MOESM2_ESM.docx]

**Appendix 2 Methodological quality appraisal**

| Qualitative studies | Item number of checklist | | | | | | |
| --- | --- | --- | --- | --- | --- | --- | --- |
|  | S1 | S1 | 1.1 | 1.2 | 1.3 | 1.4 | 1.5 |
| Iles-Smith 2005 | Y | Y | Y | Y | Y | Y | Y |
| Mitchell 2009 | Y | Y | Y | Y | Y | Y | Y |
| Lai 2012 | Y | Y | Y | Y | Y | Y | Y |
| Yu 2013 | Y | Y | Y | Y | Y | Y | Y |
| Monaro 2014 | Y | Y | Y | Y | Y | Y | Y |
| Cervantes 2017 | Y | Y | Y | Y | Y | Y | Y |
| Gullick 2017 | Y | Y | Y | Y | Y | Y | Y |
| Henry 2017 | Y | Y | Y | Y | Y | Y | Y |
| Lovell 2017 | Y | Y | Y | Y | Y | Y | Y |
| Walker 2017 | Y | Y | Y | Y | Y | Y | Y |
| Nilsson 2019 | Y | Y | Y | Y | Y | Y | Y |
| Wang 2022 | Y | Y | Y | Y | Y | Y | Y |
| Mehta 2024 | Y | Y | Y | Y | Y | Y | Y |
| Item number check list key*: S1. Are there clear research questions, S2. Do the collected data allow to address the research questions, 1.1. Is the qualitative approach appropriate to answer the research question, 1.2. Are the qualitative data collection methods adequate to address the research question, 1.3. Are the findings adequately derived from the data, 1.4. Is the interpretation of results sufficiently substantiated by data, 1.5. Is there coherence between qualitative data sources, collection, analysis and interpretation? | | | | | | | |
| Quantitative randomized controlled trials | Item number of checklist | | | | | | |
|  | S1 | S1 | 2.1 | 2.2 | 2.3 | 2.4 | 2.5 |
| Fishbane2017 | Y | Y | Y | Y | Y | U | Y |
| Item number check list key*: S1. Are there clear research questions, S2. Do the collected data allow to address the research questions, 2.1. Is randomization appropriately performed, 2.2. Are the groups comparable at baseline, 2.3. Are there complete outcome data, 2.4. Are outcome assessors blinded to the intervention provided, 2.5 Did the participants adhere to the assigned intervention? | | | | | | | |
| Quantitative non-randomized studies | Item number of checklist | | | | | | |
|  | S1 | S1 | 3.1 | 3.2 | 3.3 | 3.4 | 3.5 |
| Ma2010 | Y | Y | Y | Y | Y | U | Y |
| Cho2012 | Y | Y | Y | Y | Y | Y | Y |
| Schanz2017 | Y | Y | Y | Y | Y | U | Y |
| Shi2019 | Y | Y | Y | Y | Y | U | Y |
| Kaiser2020 | Y | Y | Y | Y | Y | Y | Y |
| Hundemer2023 | Y | Y | Y | Y | Y | Y | Y |
| Item number check list key*: S1. Are there clear research questions, S2. Do the collected data allow to address the research questions, 3.1. Are the participants representative of the target population, 3.2. Are measurements appropriate regarding both the outcome and intervention (or exposure), 3.3. Are there complete outcome data, 3.4. Are the confounders accounted for in the design and analysis, 3.5. During the study period, is the intervention administered (or exposure occurred) as intended? | | | | | | | |
| Mixed methods study | Item number of checklist | | | | | | |
|  | S1 | S1 | 5.1 | 5.2 | 5.3 | 5.4 | 5.5 |
| Cervantes2021 | Y | Y | N | N | N | U | Y |
| Item number check list key*: S1. Are there clear research questions, S2. Do the collected data allow to address the research questions, 5.1. Is there an adequate rationale for using a mixed methods design to address the research question, 5.2. Are the different components of the study effectively integrated to answer the research question, 5.3. Are the outputs of the integration of qualitative and quantitative components adequately interpreted, 5.4. Are divergences and inconsistencies between quantitative and qualitative results adequately addressed, 5.5. Do the different components of the study adhere to the quality criteria of each tradition of the methods involved? | | | | | | | |
